# Application of deep learning towards automated electromyographic wave classification for neuromonitoring in thyroid and parathyroid surgery

**DOI:** 10.1093/bjsopen/zraf158

**Published:** 2026-01-08

**Authors:** Thomas J Musholt, Petra B Musholt, Tobias Kortus

**Affiliations:** Section of Endocrine Surgery, Department of General, Visceral and Transplantation Surgery, University Medical Center Mainz, Mainz, Germany; Section of Endocrine Surgery, Department of General, Visceral and Transplantation Surgery, University Medical Center Mainz, Mainz, Germany; Chair for Scientific Computing, University of Kaiserslautern-Landau (RPTU), Kaiserslautern, Germany

## Abstract

**Background:**

Intraoperative neuromonitoring—that is, recording of electromyographic signals—is used routinely during (para)thyroid surgery. Surgeons label selected signals to document nerve identity, body side, and time point of stimulation (before or after resection), with a mislabelling rate of 20%. For the purpose of an automated error alert of mislabelled electromyographic signals, the authors developed a multitask one-dimensional convolutional neural network.

**Methods:**

Raw intraoperative neuromonitoring data were corrected using MIONQA software. Labelled electromyographic signals were extracted and metadata (duration of surgery, timing, median electromyographic peak values of actual surgery) were added to each electromyographic wave. Between 150 and 280 extracted features were used to train, validate, and test various convolutional neural networks.

**Results:**

Available raw data from a single centre including 1541 operations with continuous intraoperative nerve monitoring and 508 with intermittent intraoperative nerve monitoring between 2014 and 2024 were used. By repeated adjustments of the model architecture and the number of extracted features, an optimized one-dimensional convolutional neural network was designed. After multiple runs with randomized training (11 414 electromyograms) and test (4891) data, the final optimized convolutional neural network achieved a mean(standard deviation) accuracy of 95.72(0.76)% for correct identification of recurrent laryngeal, vagal, and superior laryngeal nerves; 97.68(0.72)% for correct prediction of the resected body side; and 97.61(0.89)% for correct identification of the stimulation time point (before *versus* after resection). The receiver operating characteristic curve for classification of the electromyographic peak signals had an excellent area under the curve of 0.993.

**Conclusion:**

The newly developed convolutional neural network enables accurate automated classification of electromyographic peak signals, facilitating the identification and correction of mislabelled intraoperative nerve monitoring data. Such optimized data quality is essential for artificial intelligence training, enabling neuromonitoring machines to alert the surgeon in the operating theatre of mislabelling. Future studies will aim to include a wider range of clinical scenarios and external data sets, in order to further optimize the existing labelling tool and allow clinical applications.

## Teaser

The newly developed deep learning model enables accurate automated classification of electromyographic signals generated by intraoperative neuromonitoring during thyroid surgery. The model distinguishes three nerves, the side, and the occurrence before or after resection with a mean accuracy of more than 95%.

## Introduction

Intermittent (i) and, to a lesser extent, continuous (c) intraoperative nerve monitoring (IONM) are used routinely in thyroid and parathyroid interventions to assess the function of the recurrent laryngeal nerve (RLN) (vocal cord function via an electromyogram derived from the vocalis muscle), the vagal nerve, and the external branch of the superior laryngeal nerve (EBSLN) during the surgical procedure. The main aim is to predict postoperative vocal cord mobility and consequently change the resection strategy during surgery in the event of an assumed unilateral vocal cord paresis, in an effort to avoid definitive bilateral paresis^1–5^. For accurate prediction of RLN function, the amplitude of the electromyographic (EMG) signal recorded at completion of (para)thyroid resection on each side of the neck is the most important parameter.

In cIONM, early intraoperative detection of impending RLN damage can help preserve nerve function. At the beginning of the neuromonitoring era in thyroid surgery, it was assumed that an amplitude value of > 100 µV after stimulation of the ipsilateral vagal nerve confirmed preserved function in almost all instances. However, with increasing experience, it became apparent that vocal cord function can be impaired or lost even when the threshold of 100 µV was exceeded^6^. Therefore, it was proposed to raise the threshold to 250 µV in order to increase false-negative predictions, which in turn raised false-positive predictions. A key study^5^ with members of the international neuromonitoring group revealed that the relationship between amplitude value at the beginning and end of a resection correlates with postoperative RLN function; if the amplitude value at the end of resection is less than 50% of the initial value, postoperative paresis can already be assumed with a low probability, even though the amplitude after resection can be much higher than 250 µV^5,7–9^. The probability of vocal cord paresis increases further with a rising gap between the initial and the postresection amplitude, until it reaches 100% (certain paresis).

When using IONM, one can state with certainty that the ipsilateral vocal cord is paralysed in the event of a persistent loss of signal (LOS) for vagal nerve stimulation (upstream of the RLN), whereas intact RLN function is assured if the EMG signal remains the same before and after resection. Between these extremes, however, there is a grey zone in which the prediction of postoperative nerve function is more uncertain and can only be estimated during the operation by the surgeon, who may be overwhelmed with the wealth of IONM information during the procedure. Nevertheless, decisions in the operating theatre depend on these IONM results, underlining the clinical need for further improvement of the accuracy of predictions based on neuromonitoring signals.

One possible solution is to process more of the information recorded by the IONM device. So far, only the amplitude and latency of nerves (RLN, vagal nerve, EBSLN) have been evaluated as parameters for the prediction of nerve function. This corresponds to only a fraction of the available recorded information. Moreover, based on the numerical values alone, it is not possible to differentiate artefacts (from contaminating sources such as bipolar coagulation) from correct EMG signals derived from the respective nerve. Therefore, it was stated in national and international guidelines^1,3^ that the EMG wave itself must be displayed—for example on the screen of the IONM device—to verify the true response of the vocalis muscle after stimulation of the RLN or vagal nerve. Comparison of consecutive EMG signals can reveal changes in EMG signal shape or drop of signal amplitudes, which may be valuable predictors of postoperative vocal cord mobility. Additionally, cIONM enables the display of trend curves for amplitude and latency that contain important additional information, such as the beginning of a slow and continuous amplitude decrease owing to traction on the RLN, by lifting a thyroid lobe during resection^4^. It is not possible for the surgeon to process this wealth of information during the surgical procedure.

Considering the issues described, improving IONM devices with deep learning models for the processing of recorded EMG data that predict postoperative nerve function appears to be a promising solution. The quality of the underlying data is of utmost importance for the training of such models. As shown previously by the authors’ group^10^, IONM data acquired during routine procedures include an error rate of about 20%. Identification and correction of these errors has so far been possible only by considerable scrutiny of data using the human eye and manual corrections. The integration of deep learning models for the evaluation of IONM data is a first step towards an automated error alert of mislabelled EMG signals, to be displayed by the IONM device. For these reasons, the authors have developed a multitask one-dimensional (1D) convolutional neural network (CNN) that can automatically label and classify EMG signals.

## Methods

### Data collection and data cleaning with MIONQA

Since 2003, iIONM and cIONM have been performed routinely during thyroid and parathyroid interventions in the Section of Endocrine Surgery, Department of General, Visceral, and Transplantation Surgery of University Medical Center Mainz (Mainz, Germany). Since 2006, IONM has been conducted with the C2, and since 2013, with the C2 Xplore devices (inomed Medizintechnik, Emmendingen, Germany). IONM data were stored on the device and transferred to another personal computer (PC) for assessment of data quality with the R application MIONQA (Mainz IONM Quality Assurance and Analysis tool), as described previously^10^. The MIONQA software is available publicly at https://ionmreference.net/downloads/mionqa-software/.

Available raw data from 1541 operations with cIONM and 508 with iIONM between 2014 and 2024 were included in this study. Patients operated on between June 2014 and May 2021 were described in a previous publication^10^. After May 2021, patients were included in the same way, and the IONM raw data were corrected according to the methods described. Continuous IONM was used routinely in thyroid resection, and iIONM in parathyroid resections. IONM was applied according to national and international guidelines including stimulation of the ipsilateral vagal and RLN before and after resection^1,3,11^. The superior laryngeal nerve was stimulated, if identified visually or with IONM mapping. The study was approved by the Ethics Committee of the Rhineland-Palatinate Medical Association (project number 2023-17300-retrospektiv).

### IONM data format and data labelling

Each EMG signal recorded encompasses a 70-ms time range with 0.05-ms intervals resulting in 1400 columns with values in millivolts. In addition, the C2 Xplore device stores the exact time of stimulation (in milliseconds), the stimulation power (milliamperes), amplitude (millivolts), onset latency values (milliseconds), minimum and maximum peak latency values (milliseconds), the stimulating channel (1 of 2) and the recording channel (1 of 4), along with two labelling columns (1 for the baseline setting and 1 for the label). Therefore, each EMG signal is encoded in a single row with 1410 columns in comma separated values format. For training of the model, only the labelled EMG signals can be used.

The label is given to a specific EMG signal manually by the surgeon or another staff member in the operating theatre by selecting labels from a predefined list available on the IONM device, or from a custom-made list. The labels determine the nerve that was stimulated (V, vagal nerve; R, RLN; S, external branch of the superior laryngeal nerve), the side (l, left; r, right), and the position (before resection, 1; after resection, 2). In the Endocrine Surgery Section, standardized labels are used (V1l, V2l, V1r, V2r, R1l, R2l, R1r, R2r, S1l, S2l, S1r, S2r).

All labelled EMG signals (19 203 in total) were summarized in a separate data table within the application MIONQA. The table was filtered for the standardized labels (16 305). The excluded labels were not used to name the nerve, position, and side of an EMG signal, but to enter information such as ‘baseline’, ‘start pausing the resection’, ‘stop pausing the resection’, ‘relevant signal reduction’, ‘change of side’, ‘non-recurrent nerve’, ‘LOS’, ‘change of strategy’, ‘artefact’, ‘preoperative paresis’, and others.

Automated error detection and manual data correction were undertaken with the MIONQA software tool^10^. As a result, the cleaned IONM training data used for deep learning consisted of almost 100% correctly labelled EMG signals.

**Fig. 1:**
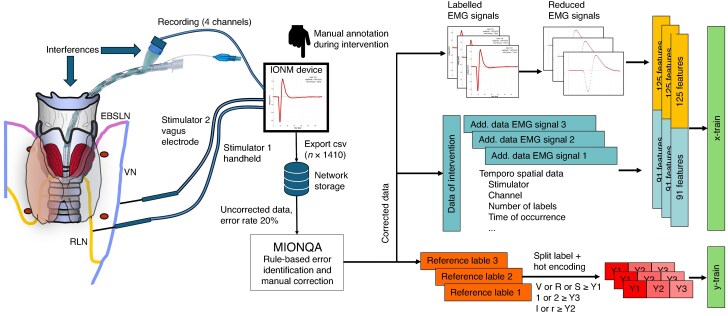
Workflow of data preparation Intraoperative neuromonitoring (IONM) was performed during thyroid and parathyroid resection. The IONM device (C2 Xplore) holds two stimulator channels. The first is usually used for intermittent IONM via a handheld probe that applies 1–2 mA to a nerve in the operative field. The second channel is used for continuous IONM via a special vagal electrode that is attached to the vagal nerve (V) and should stay in the same position during the entire resection phase of the respective side. Stimulator 2 applies 1–2 mA to the vagal nerve at a frequency of 1–2 Hz. Antegrade propagation of the stimulus leads to contraction of the vocal cord muscle in the larynx that is recorded via electrodes attached to the breathing tube. To ensure circumferential coverage of the tube, four recording channels are integrated into the adhesive tube electrode. The channel that records the highest EMG amplitude is selected automatically for storage of the EMG signal. Recording of the EMG signal can be impaired by many interferences from contaminating power sources, for example bipolar coagulation or heating devices, as well as movements of wires or the breathing tube. To document the results of IONM, the surgeon annotates selected EMG signals during surgery. All EMG signals recorded during surgery are stored on the device in a separate file with patient identifiers in the filename. The files are exported to a network drive in comma separated values (csv) format including 1410 columns for each EMG signal and the number of rows corresponding to the number of EMG signals recorded. The raw data include an error rate of about 20% referring to device errors and human labelling errors. These errors are corrected via the R-based application MIONQA, which facilitates error identification based on rules as well as the manual correction of identified errors. The corrected data serve as a basis for training and testing of the one-dimensional convolutional neural network. Only the labelled EMG signals for each operation are summarized in a single table with the tool MIONQA. Additionally, temporospatial data from the corresponding intervention are added to each EMG signal. For feature extraction, 1 in 4 of the first 25 of 70 ms of each EMG signal are used and the temporospatial data are added to serve as x-train. The reference label of the EMG signals, which is composed of three characters, is split into three labels with hot encoding resulting in Y1, Y2, and Y3 for independent output. Add., additional; EBSLN or S, exterior branch of the superior laryngeal nerve; RLN or R, recurrent laryngeal nerve.

To account for the time context in which each EMG signal was recorded, temporospatial information from the complete intervention was attached to each EMG signal as well. This information included mean amplitude values from four recording channels, calculated latency of the first peak, and up to four normalized time points for each possible standard label. To reduce the number of features and improve model throughput, the EMG signal was reduced to the first 25 ms that contained the peaks of the EMG signal, but not the subsequent time without relevant electrical activity, and only one in four values within these 25 ms were extracted. The data described were converted into an appropriate matrix using hot encoding for categorial variables and normalization functions for numerical variables (*Fig. 1*). The standard three-character standard label was split into three separate labels to enable separate prediction of the stimulated nerve, the neck side, and the position (before *versus* after resection) with a multioutput model.

### Designing deep learning model

Data preparation, definition of the model, training, and evaluation of the model on test data was done with R (R Foundation for Statistical Computing, Vienna, Austria)^12^ using, among others, the R packages reticulate^13^ as interface between R and Python 3.11, as well as tensorflow^14^, and ‘keras3^15^ for design and computation of the model.

The data matrix, consisting of 16 305 entries, was divided randomly into a training set comprising 70% of the data (11 414 signals) and a test set comprising 30% of the data (4891 signals). Some 20% of the training data (2283 signals) was used for internal validation during training. Several CNN models were evaluated using 150–280 extracted features for training, validation, and testing purposes. For the final optimized solution, 216 features served as the input layer to a multitask 1D CNN, which includes two gated recurrent unit layers^16^, two convolutional layers^17^ with batch normalization^18^ and max pooling for shared feature extraction, before being split into three output branches (*Fig. S1*). To avoid overfitting, dropout layers^19^ and regularizers (lasso regression L1 and L2) were included.

### Estimating uncertainties of deep learning model’s predictions

Monte Carlo dropout^20^ was applied during inference, yielding an approximation of the predictive posterior distribution, from which uncertainty estimates were derived. These estimates provide complementary information, indicating the confidence and reliability of individual outputs. Analogously, the final class probabilities were computed as the average oversampled class distributions. Each posterior distribution was estimated using up to 30 individual samples with varying dropout configurations. To further improve the interpretability of the raw uncertainty estimates, the uncertainty calibration scheme outlined by Gal and Ghahramani^20^ was employed, which is analogous to the approach described by Kortus *et al.*^21^. Therefore, the empirical distributions of training set uncertainty were modelled using Gaussian distributions N(μ,σ) with mean (*μ*) and standard deviation (*σ*) for each predicted output class and model branch. For scalability, both mean and standard deviation were estimated incrementally using the iterative Welford algorithm^22^ using a subset of 330 randomly selected EMG signals. The calibrated uncertainty for new test data values was calculated as the cumulative probability—the probability that an uncertainty value in the training distribution is less than or equal to the value provided. The resulting calibrated uncertainty therefore expresses how atypical the observed test–time uncertainty is relative to the training distribution.

### Training of deep learning model

The training was performed in a virtual environment with Python version 3.11^23^ and TensorFlow version 2.18.0^14^. During the training with 80 epochs, the learning rate was reduced automatically and the best model stored, which was usually after 58–65 epochs. For every output head, the cross-entropy loss between predicted and target labels was minimized using the Adam optimizer^24^. After training, the performance of the best model was evaluated on the test data. To account for sampling variability of test and training data on model accuracy, the model was trained and tested 15 times with a random distribution of independent training and test data. A receiver operating characteristic (ROC) curve analysis was performed on the test data with the R package pROC for each of the three outputs for the model (nerve, side, position) as well as after concatenation of all three outputs into a single prediction (*Fig. S2*)^25^.

## Results

**Fig. 2:**
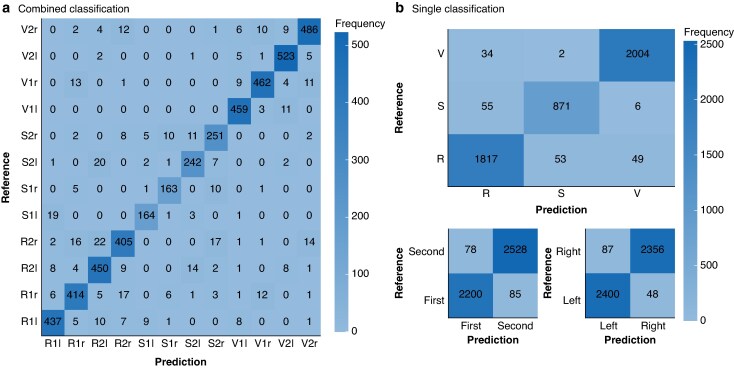
Performance of trained model on test data The confusion matrix shows the model’s performance on test data. **a** Combined classification results and **b** single classifications for nerve, side, and position. The *x*-axis represents predicted values, and the *y*-axis shows reference values. Differences between predictions and reference values are more common in distinguishing the superior laryngeal nerve from the recurrent laryngeal nerve, as well as the right recurrent laryngeal nerve from the right vagal nerve. Standardized label notation: V, vagal nerve; S, external branch of the superior laryngeal nerve; R, recurrent laryngeal nerve; r, right side; l, left side; 1 or First, before resection; 2 or Second, after resection.

The mean(standard deviations) accuracies of the multitask 1D CNN across 15 independently trained and evaluated models are summarized in *Table 1*. The accuracy of the training as well as the validation exceeded 95% for all three outputs with minimal variation between model instances. Consequently, the ROC curve analysis revealed an overall area under the curve (AUC) for the concatenated three predictions (nerve, side, position) of the test data of 0.993. The confusion matrix of predictions and reference labels for the test data showed that differences persist (*Fig. 2*). Detailed examination of these differences showed that they are primarily attributable to three causes: unusual clinical scenarios in which, for distinct reasons, the standard algorithm of stimulations (for example labelling for the left side; V1l, R1l, S1l, R2l, V2l, S2l) was not adhered to; iIONM procedures with insufficient (< 3 per side) labelled EMG signals; and EMG signals that are in the overlapping area of the signal classes, for example SBSLN and RLN.

**Table 1:** Model output from 15 training runs

	Accuracy (%)
**Output nerve**	
Training	96.53(0.67)
Validation	95.72(0.76)
**Output side**	
Training	97.80(0.62)
Validation	97.68(0.72)
**Output position**	
Training	97.49(0.96)
Validation	97.61(0.89)

Values are mean(standard deviation). The model was trained and evaluated 15 times on independent training and validation data to account for random effects when sampling the data.

**Fig. 3:**
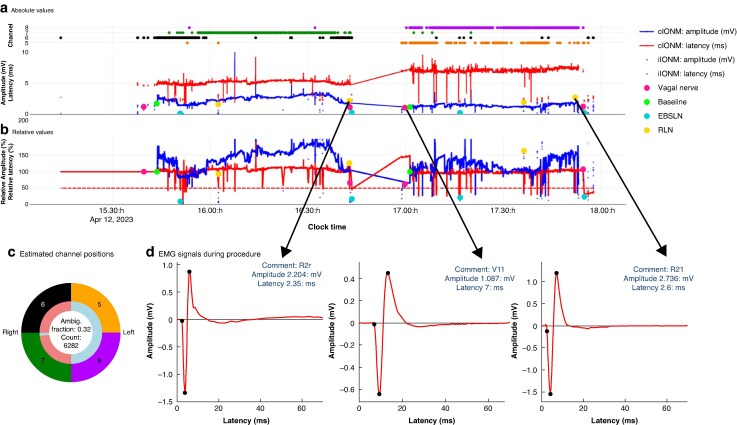
cIONM data recorded during thyroidectomy The figure shows the complete intraoperative nerve monitoring IONM data recorded during thyroidectomy. The figure was created using MIONQA software. **a** Absolute and **b** relative values for electromyographic (EMG) signal amplitudes and latencies. Lines represent continuous (c) IONM, whereas dots indicate intermittent (i) IONM. EMG signals labelled during surgery are marked with larger coloured dots, corresponding to stimulation of the vagal nerve, recurrent laryngeal nerve (RLN), and the external branch of the superior laryngeal nerve (EBSLN), both before and after resection of a thyroid lobe. The absolute latency (red line in **a**) clearly demonstrates that the right vagal nerve was recorded first, followed by the left, as indicated by the distinct latency differences between right and left vagal nerve stimulation. **c** The four channels used to record EMG signals. It is evident that channels 6 and 7 primarily captured signals from the right side, whereas channels 5 and 8 recorded signals from the left. Based on latency-dependent assignment, the position of the adhesive electrodes—or, more specifically, the corresponding channels—on the ventilation tube can be inferred. Although differentiation of right and left vagal nerve signals is generally possible owing to their latency differences, EMG signals from the RLN and the EBSLN cannot be assigned to a specific side based on EMG characteristics alone. **d** Illustration of this limitation showing three EMG signals recorded at different stages of the procedure. A reliable side assignment and evaluation of stimulation before and after resection are only feasible through temporal correlation and comparison with other EMG signals. Incorporating this contextual information into the deep learning model has significantly improved predictive accuracy. Ambig., ambiguous; R2r, recurrent laryngeal nerve after resection, right side; V1l, vagal nerve, before resection, left side; R2l, recurrent laryngeal nerve after resection, left side.

The latency of the EMG signal is one of the most important pieces of information for classification. The mean(standard deviation) of onset latency of the RLN was 2.5(0.7) ms, whereas the onset latency of the right vagal nerve was 4.2(0.8) ms^10^. In special situations, however, the latency of the recurrent nerve can be prolonged and the latency ranges from 1.1 to 10 ms, so that the signal from the RLN can look very similar to a signal from the vagal nerve, and differentiation is almost impossible, even for an experienced surgeon. Furthermore, it is not possible to assign an EMG signal from the RLN to the left or right side based solely on the signal shape. Only the context of the complete time information and comparison with reference EMG signals from the same intervention enables more accurate labelling. Continuous neuromonitoring facilitates the identification of EMG signals from the left and right vagal nerves, which also improves the prediction for the assignment of signals from the RLNs to the left or right side. For iIONM with only one or two EMG signals, a high degree of uncertainty remains (*Fig. 3*).

**Fig. 4:**
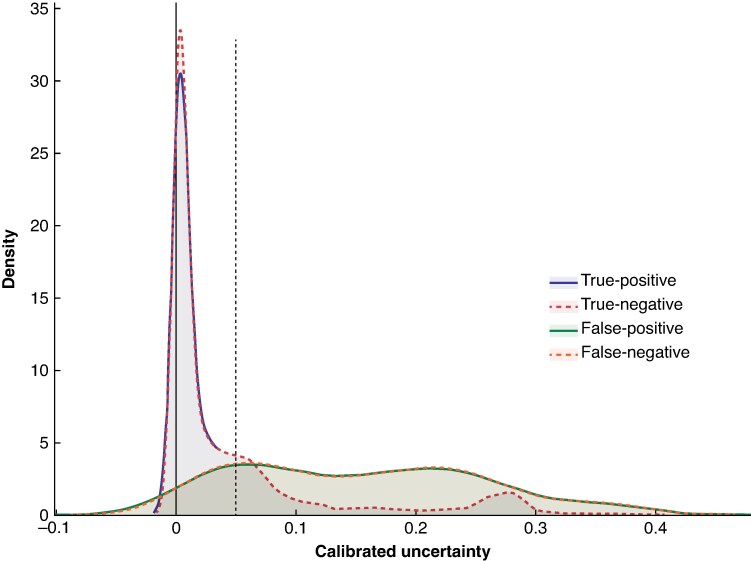
Distribution of calibrated uncertainty estimates obtained for categories true-positive, true-negative, false-positive, and false-negative Density plot illustrating calibrated uncertainties in relation to prediction accuracy for each classification category nerve. The areas for true-positive and true-negative as well as false-positive and false-negative largely overlap, making them difficult to differentiate; nevertheless, all four categories are included. The plot indicates that low uncertainty values correspond to a higher probability of true-positive and true-negative outcomes. A threshold represented by the dashed line can be used to filter predictions with high uncertainty and further improve prediction quality.

Calibrated uncertainty estimates, separated into true-positive, true-negative, false-positive, and false-negative classifications, are visualized as a density plot in *Fig. 4*, revealing moderate separation between true and false classifications. Misclassified instances tend to exhibit higher uncertainty values than correctly classified samples. This pattern suggests that, for the proposed application, uncertainty estimates provide meaningful information about the reliability of individual predictions. Consequently, uncertainty estimates can be leveraged to establish thresholds for identifying significant instances of incorrect classifications (both false-positives and false-negatives) that require additional manual review, ultimately improving the trustworthiness of the system (*Fig. 4*).

## Discussion

The findings of this study have demonstrated the feasibility of using deep learning tools for the automated classification of EMG signals in IONM during thyroid and parathyroid procedures. By leveraging a multitask 1D CNN, it was possible to achieve high classification accuracy across key parameters: nerve identity, body side, and stimulation time point. The final optimized model demonstrated consistently robust performance, achieving mean accuracies exceeding 95% across all classification tasks. Additionally, it attained an AUC of 0.993, underscoring its robustness and reliability. This study was restricted to thyroid and parathyroid interventions; however, based on their own experience, the authors can state that EMG signals do not differ if the vagal nerve is stimulated during parathyroid, thyroid, carotid or cervical oesophageal surgery. The method is therefore applicable to any intervention that uses IONM with stimulation of the cervical vagal nerve and RLN.

At this stage of the study, the model was incorporated into the data correction pipeline of the MIONQA software, which addressed the 20% mislabelling rate in manually annotated IONM data. Accurate labelling is a crucial prerequisite for the development of deep learning models, because incorrect labels introduce noise that can degrade model performance. The ability of the CNN described here to identify potential errors that can be corrected manually is a critical step toward improving data quality as a prerequisite for the development of improved and more comprehensive deep learning models. The previous rule-based algorithms used in MIONQA to identify labelling errors were dependent on amplitude and latency values measured by the devices. In particular, latency values, which are the most important feature for classification of EMG signals, can be incorrect and impair data quality analysis. Using the EMG signal within the model eliminated reliance on latency values specified by the device. This approach markedly enhanced accuracy compared with relying solely on latency values. However, achieving 100% accuracy is unrealistic. Even if the false-positive and false-negative results are low, they accumulate for the three predictions. An error rate for each prediction of 2–3% will identify about 200 signals with potential errors in a group of 20 000 signals. Therefore, human review of potential errors is still necessary at this point, and uncertainty estimates can provide additional guidance for this task.

The misclassifications in the test data were primarily attributed to three factors: deviations from standard stimulation protocols owing to unusual clinical scenarios; limited data availability in iIONM procedures; and signal overlap between different nerve stimulations, particularly between the EBSLN and RLN, or between the right RLN and right vagal nerve. These limitations highlight the need for further refinement, including the incorporation of additional contextual features, such as the identification of reliable reference signals within a surgical procedure and the elimination of artefacts to improve classification in challenging cases.

To quantify the uncertainty of predictions, Bayesian methods were applied, replacing the point estimates of traditional deep learning with a sampled predictive posterior distribution accounting for the uncertainty in the model parameters. Uncertainty estimates are an essential component for improving the quality of predictive models for safety-critical applications^20,26,27^ and have been used successfully for IONM^21^. This approach enabled a more nuanced evaluation of classification confidence and facilitated the identification of threshold values to further enhance prediction quality. The analysis of calibrated uncertainties revealed that certain misclassifications exhibited higher uncertainty levels, suggesting that uncertainty estimates could be leveraged as an additional safeguard in clinical decision-making. Yet, the limited separation of outputs of low and high uncertainties requires further improvements in both model architecture and uncertainty estimation.

A limitation of the study is that the training data originated from a single centre leveraging an IONM device from the manufacturer inomed. In view of the numerous possible factors influencing the results of IONM, such as surgeon, anaesthetist, and environment in the operating theatre, it cannot be assumed that the trained model will also provide the same results for data from other clinics or for data recorded with devices from other manufacturers. However, because iIONM and cIONM data from many different clinical scenarios were included, and considering the analysis of the impact of single features on the model’s performance, the authors believe that similar results can be achieved with data from other clinics. With respect to data recorded with devices from other manufacturers, it must be noted that the format of the data must fit the format expected by the model. In theory, it should be possible to format the data appropriately, but the authors have not been able to test this owing to lack of availability of raw data files from other devices.

Comparing the findings of the present study with those in existing literature has revealed that the deep learning model for EMG signal classification in IONM described here achieves notably high-performance metrics. A PubMed search with the keywords ‘deep learning’ and ‘thyroid’ and ‘neuromonitoring’ or ‘IONM’ revealed no results. A more detailed internet search revealed one feasibility study^28^ that applied a deep learning model for automated classification of free-running EMG waveforms during cIONM based on data from five patients. Another publication^29^ referred to the classification of compound EMG signals recorded with surface electrodes. Therefore, to the best of the authors’ knowledge, this is the first publication describing the use of deep learning models to classify EMG signals resulting from supramaximal stimulation during thyroid surgery.

In the broader context of 1D biosignal analysis, deep learning models have been applied to various signal types, including electrocardiography, photoplethysmography, and electroencephalography. A comprehensive survey by Ganapathy *et al.*^30^ in the *IMIA Yearbook of Medical Informatics* 2018 reviewed deep learning techniques for biosignal analysis, highlighting the successful application of CNNs in these domains. However, the survey noted that biomedical signal analysis had yet to fully benefit from deep learning approaches, indicating room for advancement in this field.

In the realm of time series classification, Fawaz *et al*.^31^ conducted an extensive review, presenting an empirical study of recent deep neural network (DNN) architectures. Their findings demonstrated that certain DNN models achieved high accuracy across various time series data sets, underscoring the potential of deep learning in this area.

In summary, the outcomes of the present study are consistent with the high-performance metrics reported in the literature for deep learning applications in biosignal analysis and time series classification. This work not only reinforces the efficacy of deep learning in these domains but also introduces novel methodologies tailored to the specific challenges of EMG signal classification in IONM. Future models can be developed to: discriminate artefacts from true EMG signals; minimize labelling errors of EMG signals; enhance the labelling of EMG data by automated annotations; improve the prediction of postoperative nerve function during iIONM and cIONM; and improve the identification of impending nerve damage during cIONM.

To achieve these goals, future work will focus on expanding the data set to include a wider range of clinical scenarios; including other clinics and their settings; further optimizing the existing model architecture; and developing additional models. In addition, real-time use and integration with IONM devices should be feasible. The successful development of deep learning models will ultimately improve patient safety and surgical outcomes. The complete source code of the MIONQA tool, including the deep learning model, is available at https://ionmreference.net/downloads/mionqa-software/ as installation file for Windows^®^ (Microsoft, Redmond, WA, USA) PCs. The download page also includes video tutorials on use of the application.

## Supplementary Material

zraf158_Supplementary_Data

## Data Availability

The data used in this study form part of other ongoing research projects within the authors’ group and identified data are therefore not available at this time. The authors are considering sharing anonymized data at a later date upon reasonable request.
