# Supplementary material for: Application of deep learning towards automated electromyographic wave classification for neuromonitoring in thyroid and parathyroid surgery
Source: BJS Open. 2026 Jan 8;10(1):zraf158. doi: 10.1093/bjsopen/zraf158 (PMC12781199; doi:10.1093/bjsopen/zraf158)
Supplement: zraf158_Supplementary_Data [file zraf158_supplementary_data.docx]

**Application of deep learning towards automated EMG wave classification for neuromonitoring in thyroid and parathyroid surgeries**

Thomas J. Musholt1 (ORCID ID: 0000-0002-4498-7439), Petra B. Musholt1 (ORCID ID: 0009-0004-3398-7283), Tobias Kortus2 (ORCID ID: 0000-0002-0987-8544)

^1^ 1. Section of Endocrine Surgery, Department of General, Visceral and Transplantation Surgery,

University Medical Center Mainz, Mainz, Germany

^2^ 2. Chair for Scientific Computing, University of Kaiserslautern-Landau (RPTU), Kaiserslautern, Germany

**Corresponding author.**

Univ.-Prof. Thomas J. Musholt, M.D., FEBS-Endocrine Surgery

Head, Section of Endocrine Surgery

Department of General, Visceral and Transplantation Surgery

University Medical Center Mainz

Langenbeckstr. 1

55131 Mainz, Germany

**ORCID ID: 0000-0002-4498-7439**;

**Supplementary Materials - Index**

| **Supplementary Figures and Tables** |  |
| --- | --- |
| **Supplementary Figure 1: Optimized 1D CNN** l | *page 2* |
| Supplementary Figure 2: ROC analysis of for the aggregated predictions on test data | *page 3* |

**Supplementary Figures and Tables**

**Supplementary Figure 1: Optimized 1D CNN**

**
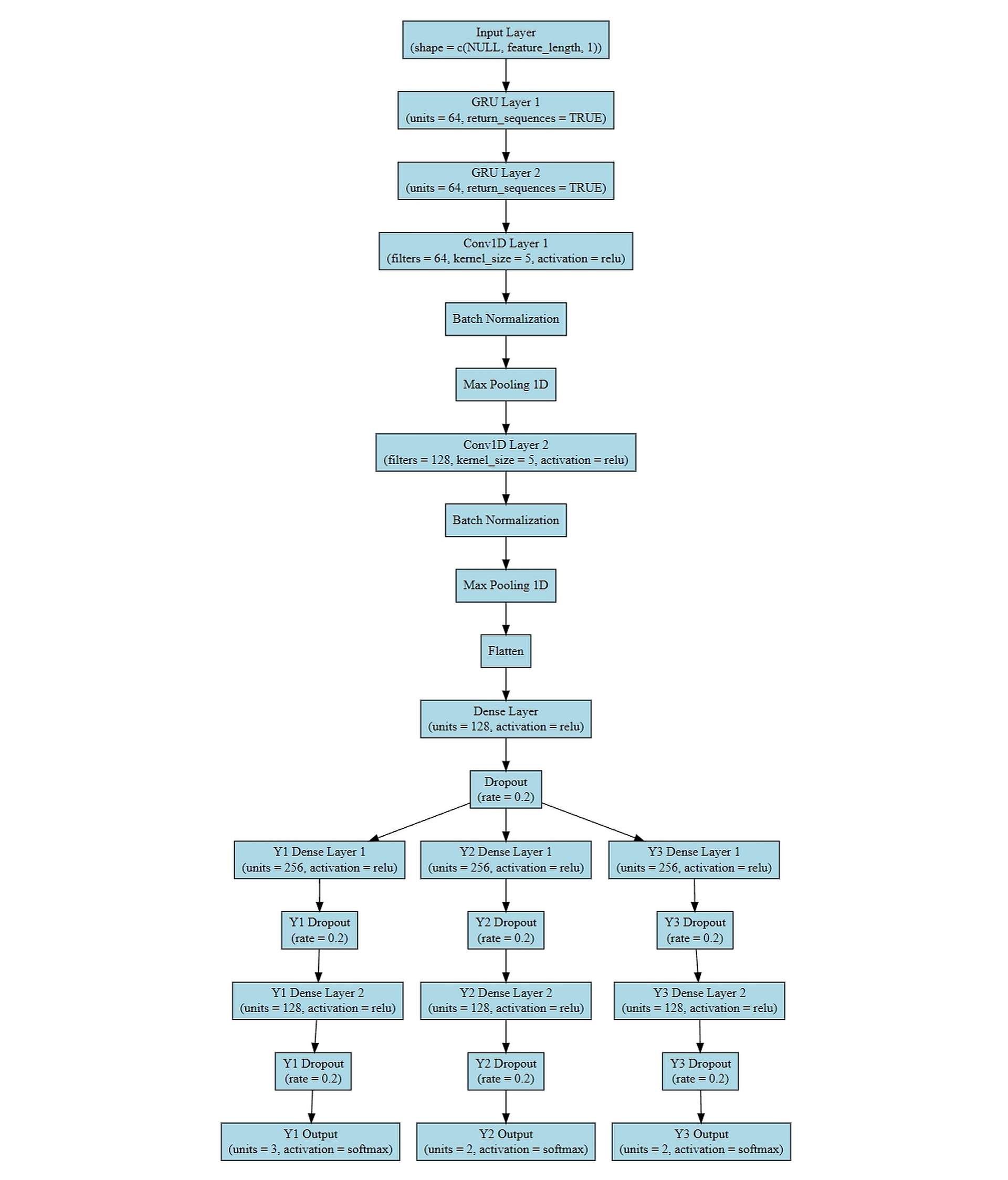
**

The multitask 1D CNN includes two Gated recurrent unit (GRU) layers and two convolutional layers with batch normalization and max pooling for shared feature extraction, before being split into three output branches. To avoid overfitting dropout layers and regularizers (l1 and l2) were included. The first branch of the model has 3 outputs referring to the classification of the nerve (R, S, V) while the second and third branch of the model have two outputs for the side (left and right) and the position (1 and 2).

**Supplementary Figure 2: ROC analysis of for the aggregated predictions on test data**


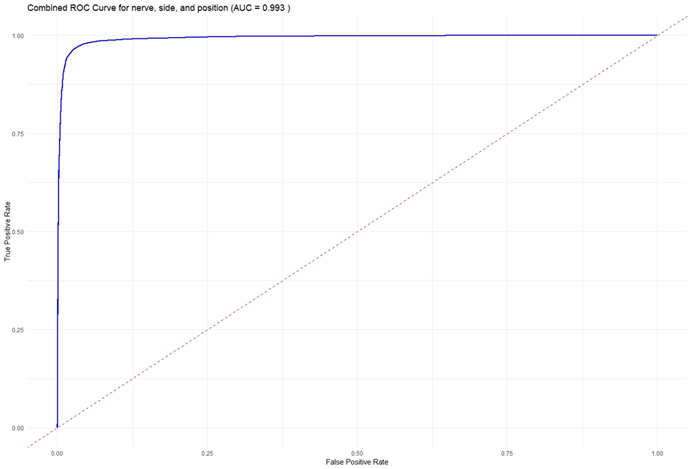


The receiver operating characteristic (ROC) diagram illustrates the false positive rate on the x-axis and the true positive rate on the y-axis for the aggregated predictions on test data. The area under the curve (AUC) is calculated to be 0.993, indicating the model's high accuracy.
